# Finding uncommon ground: Extremist online forum engagement predicts integrative complexity

**DOI:** 10.1371/journal.pone.0245651

**Published:** 2021-01-19

**Authors:** Andrew L. Gregory, Paul K. Piff

**Affiliations:** Department of Psychological Science, University of California Irvine, Irvine, California, United States of America; Lancaster University, UNITED KINGDOM

## Abstract

How do interactions with an ideologically extreme online community affect cognition? In this paper, we examine whether engagement with an online neo-Nazi forum is associated with more one-sided, “black and white” thinking. Using naturalistic language data, we examined differences in integrative complexity, a measure of the degree to which people acknowledge and reconcile conflicting ideas and viewpoints, and contrasted it with Language Style Matching, a measure of group cohesion. In a large web scraping study (*N* = 1,891), we tested whether two measures of engagement and interaction with the community are associated with less complex, balanced cognition. Using hierarchical regression modeling, we found that both individuals who had been community members for longer and those who had posted more tended to show less complexity in their language, even when accounting for mean differences between individuals. However, these differences in integrative complexity were distinct from group cohesion, which actually decreased with our measures of engagement. Despite small effect sizes, these findings indicate that ideologically extreme online communities may exacerbate the views of their members and contribute to ever-widening polarized cognitions.

## Introduction

Online social spaces have a great power to unify. People with fringe interests and beliefs, isolated before the technological boom at the end of the 20^th^ century, can now be united in online affinity groups, a trend with mixed social consequences. On the one hand, cohesive online movements can move from the internet offline, organize for genuine social change, and change the modern political lexicon [1]––for example, the Occupy Wallstreet movement spread throughout the United States, increasing the amount of public discourse about income inequality [2]. On the other hand, there are countless instances of extremist groups using the internet to organize violence, from the Islamic State’s prolific online campaigns to the tragedy at the Unite the Right Rally in Charlottesville, Virginia that resulted in a state of emergency declaration and homicide [3]. These groups unite those with similar extreme values, but their effects on members’ attitudes and beliefs are still largely unknown [4]. Can these groups exert social influence on their members and further radicalize them? In this research, we use computational linguistic markers of cognition to examine how thought and communication become more polarized within an extremist online forum over time.

### Online ideological silos

In general, the internet increases the diversity of political information and news sources that people consume [5, 6]. On Twitter, Barberá and colleagues found that retweeting opposing political sources was frequent, even if the information is ideologically charged [7]. However, metaphorical “echo chambers”—an environment in which similar ideas are repeated and amplified––are a contentious issue within social and political debates [8]. Echo chambers are seen as a modern threat in part due to their potential for allowing individuals to silo themselves off from differing information and viewpoints, a dynamic that could heighten bias, misinformation, and polarization [9].

Extremist online groups may embody the echo chamber that the news media and the politically-minded fear, in that they provide a social space where similar extreme views can be shared, repeated and amplified [10]. Various factors may contribute to this. First, members are a self-selecting population. For example, Stormfront—the neo-Nazi online forum examined in this research—features swastikas, racial slurs, and headlines that would likely scare off any casual readers. Therefore, members are likely to have similar views on their central values (e.g., Caucasian superiority).

In addition, Stormfront is strict about its treatment of diverse viewpoints; dissenting voices are actively siloed into a sub-forum termed “Opposing Views Forum.” If an “opponent”—anyone who is against white supremacy or identifies as non-white—posts in another sub-forum, their post is flagged and moved to “Opposing Views.” After multiple infractions the user may be banned. Not only is the forum appealing to one specific population, but anyone who directly opposes and argues against the dominant ideology can be removed, keeping the forum ideologically homogenous. Despite national news media coverage that might attract opposition to their discussions, discourse is limited to members who generally agree about the supremacy of white Europeans by filtering posts from individuals that moderators deem “here to argue” [11].

On the other hand, the homogeneity of the information on the site may increase the social influence to conform to the beliefs and behavior of others, leading to extreme, polarized views [12–14]. Consensus and cohesion of a group is what Janis [15] found to be the most important precursor for “groupthink”—the mode of thinking that occurs when people are “deeply involved in a cohesive in-group, when the members’ strivings for unanimity override their motivation to realistically appraise their alternative courses of action.” Among other symptoms, groupthink causes members to self-censor their ideas that deviate from the norm, question the group less, and perceive more unanimity than there actually is, leading to irrational and dysfunctional outcomes [16].

The social influence that emerges within an extreme and actively homogeneous group may be even more pernicious online due to its anonymous setting, without face-to-face interactions, real names, and ostensible personal accountability. Anonymity and deindividuation—the loss of individualism that occurs under conditions of anonymity—can lead individuals to engage in antisocial behavior like cyberbullying [17], aggression [18], and out-group discrimination [19]. Anonymity in digital communications also increases social identification with salient groups; deindividuation leads people to identify more with their in-group [20] and can amplify social influence [21]. In this way, members of online communities may see themselves less as an individual and more as a group member, which can lead to the adoption of group attitudes that diverge from or are more extreme than one’s own viewpoints.

Together, this conceptual overview and empirical analysis indicates that in online extremist communities, polarization may occur through the initial self-selection of the membership and the active filtering of dissent, both of which should heighten ideological homogeneity. At the same time, social cohesion with the group may increase, potentially leading to deindividuation and increased aggression arising from anonymity. Here, we examine whether engagement in online extremist communities is associated with more narrow-minded, simple cognition using the linguistic marker of integrative complexity. At the same time, we study the relationship between engagement and a linguistic marker of group cohesion, Language Style Matching, in order to see if polarization co-occurs with increased group cohesion.

### Integrative complexity

Language can provide direct insight into changes in cognition and attitudes over time. By codifying the types of words a person uses, quantitative linguistic analyses can be used to approximate a variety of psychological constructs, including a person’s emotions and moral concerns [22, 23]. For example, in a study by Pennebaker [24], the words in trauma victims’ journals were counted by their function. Results indicated that as people processed their trauma, they used more causal words to explore the context and reasons behind their histories, which in turn predicted health outcomes.

Most pertinent to the current investigation is cognitive complexity, a constellation of constructs that indexes individuals’ simplicity versus complexity of thought. Specifically, we focus on “integrative complexity,” which refers to the extent to which individuals assimilate and differentiate opposing ideas and concepts in their language [25]. If a sentence is simple or uses words associated with certainty, like “The car is (definitely) black,” it would score low (1) on integrative complexity [26]. By contrast, insofar as uncertainty and contrasting ideas inhabit the same sentence, for example “The car is black, but it has a white stripe,” it would yield a mid-range score (3) for complexity. An even more integratively complex passage shows not only differentiation, but integration, in which alternatives and their potential interactions are explored, as in “The car’s black paint in conjunction with its white stripe makes it look like a skunk.” As integrative complexity increases, it signals both the recognition of other points of view and an effort to reconcile them. Accordingly, integrative complexity can serve as an indicator of groupthink [27]. Engaging in groupthink has been associated with decreases in integrative complexity, when alternatives are not explored. Integrative complexity then rebounds after the period of groupthink is over and balanced discussion resumes.

Research has examined the sequelae of cognitive complexity in the geopolitical realm. For example, integrative complexity is associated with the outcomes of international conflict: in analyses of diplomatic communications, integrative complexity decreases in crises that end in war, but remains stable as a peaceful resolution is found [28]. Similarly, after a surprise terrorist attack, the leadership of the victim country shows a drop in integrative complexity, suggesting a moral-minded motivation to oppose and dismiss enemies’ viewpoints instead of a more complex, compromising stance [29]. These findings indicate that thinking and language balances multiple perspectives in successful negotiations but become simpler and more one-sided as entities fall in opposition. On the other hand, integrative complexity has been shown to increase after exposure to other cultures. For example, Tadmoor and Tetlock [30] found that people who have lived abroad and are oriented toward more than one culture tend to process information more complexly, driving creativity and unconventional problem solving [31].

Together, the work we have described indicates that integrative complexity can serve as a marker of ideological conviction and be shaped by social and situational pressures. Extending this research, we examine patterns of polarized cognition in an ideologically extreme White Supremacist online community, specifically examining whether integrative complexity decreases as community engagement increases. To strengthen our understanding of the dynamics of the forum, we also calculate changes in group cohesion through language usage and examine whether these shifts are distinct from integrative complexity.

### Language style matching

Central to the discussion of extremist communities is not only members’ ideological indoctrination, but the unification and cohesion of a group. A unified group is powerful, both in the social influences like deindividuation that occur within members and the strength in numbers of a united force working together. Cohesion also presents in language usage; when a dyad or group becomes more cohesive, deindividualized, and understanding of one another, they adopt a similar linguistic style [32]. Independent of the nouns and verbs that make up the bulk of meaning and content, language can be compared on use of function words—the words that make up the semantic structure of the sentence. Quantifying the similarity of texts can be done through language style matching (LSM), which calculates the frequency of nine types of function words (e.g., pronouns, prepositions, and conjunctions) [33]. Those frequencies are compared between dyads, with each relationship receiving a score that indicates similarity between the texts. Linguistic similarities as measured by LSM are valuable indicators of cohesion and understanding, as people naturally mimic and assimilate to others’ speaking styles when they are in sync. For example, LSM scores predict the success of romantic relationships [34], with more similar language preceding the desire for a second date after an initial encounter. Most relevantly, LSM is associated with cohesiveness of groups in computer mediated interaction [35] and with more emotional support in stressful conversations [32].

Accordingly, through LSM we will test whether engagement is related to increased group cohesion, in addition to polarized, one-sided cognition. When explored in concert, LSM and integrative complexity together have the potential to provide more nuanced insights into the social dynamics within the group than either variable on its own. For example, if polarization increases with engagement, our analysis of LSM may be able to show that this shift occurs alongside the strong social influence of cohesion. On the other hand, if language similarity decreases while polarization increases, polarization is in opposition to group norms and social influences like cohesion or deindividuation that might bring members to write in similar ways.

### Current research

In this research, we examine integrative complexity as a function of engagement with an online extremist White Supremacist forum: Stormfront.org. We hope to ascertain the extent to which the individual’s cognition becomes less balanced and more group-focused, conflict-oriented and one-sided. We hypothesize that as members engage with this extremist ideological forum and are exposed to the views therein, as measured by time spent as a member and the number of posts made, they will exhibit less integrative complexity in their verbal behavior online (i.e., their forum posts). We will also explore the data using language style matching as a measure of group cohesion. As engagement increases, we hypothesize that LSM will increase, indicating stronger identification with the group. Data are publicly available via the Open Science Framework (https://osf.io/6udrv/?view_only=f0985003d6e04a94b80d0934c2edbdea).

## Method

This study consists of data from the StormFront “Ideology and Philosophy” sub-forum, gathered in February 2018. Specifically, we wrote a web scraping application in the *R* programming language to collect post data and modeled integrative complexity using two hierarchical linear regressions that estimate the effects of engagement on integrative complexity. The “Ideology and Philosophy” was chosen for its combination of both size and subject matter. The sub-forum has the third most posts on the website. Two sub-forums are more popular, but they are not suitable for our research question: “News”—in which headlines and links to articles are posted but not original content—and “Lounge” where members can socialize and meet each other. We chose this specific sub-forum because it is the most popular on the site that relates to the changes in worldviews and extremism pertinent to this study.

### Scraping

To gather linguistic data from the forum, we wrote a script using the R programming language and the *rvest* package to read html data. The scraper collected data from the ideology forum, which had 21 pages of posts at the time of data collection. Each page contained links to posts that had up to 178 replies. Because of the controversial and fringe nature of the group, the website was equipped with protection that limits the number of requests from a certain internet protocol (IP) address. This is intended to thwart distributed denial-of-service (DDoS) cyberattacks that could disable the site, but it also limited the rate of our navigation through the website. Despite not violating the terms and conditions of the site—and including a half-second temporal delay after collecting data from each page—we still experienced rejections from the server. After each rejection, we restarted the program. In total, the scraper gathered data from 1,922 unique posts from October 17, 2017 to February 7, 2018. At the time of data collection, there had been 338,018 posts in the history of the “Ideology and Philosophy” subforum. This means that our sample represents .57% of the total posts on the website. However, because posts are archived and not directly navigable after six months, the number of observations that were available on the sub-forum at the time of scraping was 4,014; our sample represented 47.8% of the available data on the forum.

#### Post data

Each observation includes information specific to the post, including: (a) the text from the post, (b) the date and time of the post (in Unix timecode), and (c) the url of the post. Each observation also includes information about the member who posted, including: (a) his or her unique identification number, (b) user name, (c) self-described location, (d) date joined, and (e) the number of times the member posted at the time of data collection––our first measure of engagement. As our second measure of engagement (deemed “time to posting”), we calculated the difference between the date of the posting and the join date in order to calculate the amount of time that the author had been a member of the forum by the time of posting. In addition, we calculated the number of words of the post because word count is correlated with integrative complexity and thus represents an important co-variate [36]. The forum takes the quality of its postings seriously; in the information posted for new members, they state that posts are flagged for improper grammar, typos, excessive capitalization, spam, and quoting news media. Forums are strictly moderated, which the organizers attribute in part to the lawsuits that the website faces when members break copywrite laws by reposting news stories, and hate speech laws when members engage in personal attacks or use racial epithets [11]. To post on the forum, members must be registered with an email address and pass a “captcha” automation check. After registration, members have all of their posts read and approved by a designated moderator before they are published until they have gained a certain level of trust on the site. These precautions effectively eliminate the possibility that posts were bot-generated. For these reasons, we opted not to attempt to filter any posts even if they do not look user-generated.

### Integrative complexity

Each post was analyzed using the online Automated Integrative Complexity system [36]. Coding integrative complexity by hand is the most accurate way of scoring the construct, but it is time-intensive, requires extensive training, and is subject to human error and judgment. The online automated tool is a validated and reliable way of measuring the construct using key words and phrases, showing an *alpha* of .72 on a standard test document, just below .85 of human scorers [36]. Practical for studying large datasets, the program scores each passage on integrative complexity along with an assortment of subindices [37]. Integrative complexity is scored on a scale of 1 to 7, with higher values signifying more differentiation and integration of disparate ideas.

### Language style matching

Each post was analyzed using the *LIWCalike* package for *R*, which counted the occurrence of a variety of word types. Of interest were nine types of function words: personal pronouns (e.g., I, his), impersonal pronouns (e.g., another, someone), auxiliary verbs (e.g., might, would), articles (e.g., the, a), common adverbs (e.g., always, naturally), prepositions (e.g., of, in), conjunctions (e.g., and, because), negations (e.g., no, never), and quantifiers (e.g., much, few). LSM of each function word (FW) was calculated as the similarity between each post (p) and the rest of the dataset (d). The absolute value of the difference between the proportion of the function word in the post text and the proportion of the function word in the rest of the posts in the corpus is divided by the sum of the two proportions plus a marginal number to prevent zero in the denominator. This is then subtracted from one, shown in the formula below:

$${LSM}_{FW}=1-\frac{\left|{FW}_{p}-{FW}_{d}\right|}{{FW}_{p}+{FW}_{d}+.0001}$$

This calculation yields a score bounded by zero (very dissimilar) and one (very similar), which is then averaged over the nine different function word types to achieve the LSM score.

## Results

Of the 1,922 observations collected, 31 were dropped because the post contained no words (e.g., some posts contained only an image), leaving 1,891 observations from 432 individuals. The mean join date was Sunday, August 25, 2013, with a standard deviation of 1,795.63 days (or 4.91 years). The mean amount of time from joining to each posting was 1,558.15 days (*Median* = 728.52 days, *SD* = 1,794.30 days, or 4.91 years, *Min* = 16.40 hours, *Max* = 16.43 years), and the mean number of posts that each member had written was 4,297.08 (*Median* = 905, *SD* = 8,331.51, *Min* = 1, *Max* = 75,953). The mean words per post we collected was 125.22 (*Median* = 51, *SD* = 315.64, *Min* = 1, *Max* = 4,828). Integrative complexity was generally low, with a mean of 1.98 (*Median* = 1.5, *SD* = 1.18), suggesting moderate differentiation of contrasting ideas, but very little integration of those ideas. Language Style Matching scores had a mean of .61 (*Median* = .69, *SD* = .25), relatively low compared to other work from Gonzales et al., which examines computer mediated communication within groups (*M* = .88) [35].

### Integrative complexity

#### Number of posts

**Fig 1:**
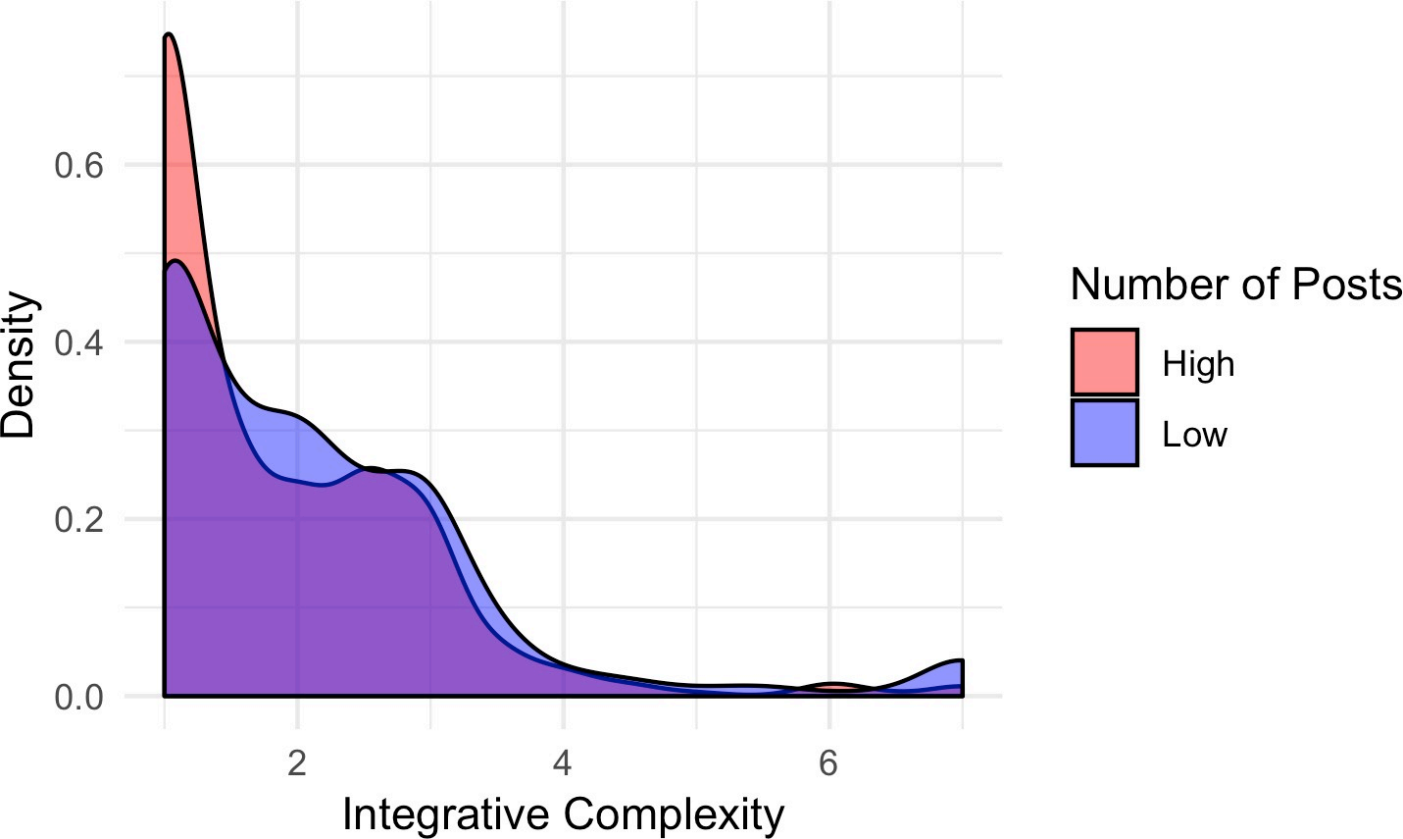
Density of integrative complexity scores split by author’s number of posts. Density of integrative complexity scores for posts by authors who have posted more or less than the sample median (905), shown in red and blue, respectively.

In a between-subjects hierarchical linear regression, integrative complexity was modeled as a function of the number of posts by the member and number of words in the post (*N* = 1,891). We used a fixed-effects model, accounting for individual differences in complexity while estimating the relationship of interest as constant across all subjects. Although a random-effects model may also have been applicable, our research question primarily concerns the effect of forum engagement across all members and not how that effect varies by member. In addition, we were unsure about unobserved variables that might bias our results. To best account for unobserved variables, a fixed effects model can be used, which allows each individual to serve as their own control [38]. For each member, a unique intercept was estimated to account for the lack of independence of observations. Independent variables were standardized for best estimation using the *lme4* and *lmertest* packages in *R*. As shown in Table 1 below, standardized number of posts was negatively associated with integrative complexity (*β* = -.08, *t*(181.79) = -2.99, *p* = .003, 95% *CI* [-0.14, -0.03]) when adjusting for word count. Members who wrote more posts on the forum tended to show less integrative complexity (see Fig 1). An increase of 8,331.51 posts was associated with a .08 decrease in integrative complexity when adjusting for word count and individual mean differences, a very small effect.

**Table 1:** Integrative complexity as a function of number of posts and time since joining the forum.

	*β*	*95% CI*	*p*
Model 1			
(Intercept)	1.97	1.92 – 2.03	< 0.001***
Number of Posts (std)	-0.08	-0.14 – -0.03	0.003**
Words (std)	0.81	0.76 – 0.86	< 0.001***
Random Effects			
σ^2^	0.69		
τ_00_	0.08		
ICC	0.10		
Marginal R^2^ / Conditional R^2^ = 0.464 / 0.520			
Model 2			
(Intercept)	1.98	1.92 – 2.04	<0.001***
Time to Post (std)	-0.05	-0.12 – 0.00	0.076 †
Words (std)	0.76	0.71 – 0.81	<0.001***
Random Effects			
σ^2^	0.71		
τ_00_	0.08		
ICC	0.10		
Marginal R^2^ / Conditional R^2^ = 0.468 / 0.522			
Model 3			
(Intercept)	1.98	1.92–2.03	<0.001***
Time to Post (std)	-0.01	-0.08 –.05	0.59
Number of Posts (std)	-.08	-.15,–.01	.01**
Words (std)	.76	.70 –.81	<0.001***
Random Effects			
σ^2^	0.71		
τ_00_	0.07		
ICC	0.09		
Marginal R^2^ / Conditional R^2^ = 0.477 / 0.526			

Note: Random effects refer to intercepts estimated for each member.

† p< .1

* p < .05

** p < .01

*** p < .001.

#### Time to post

In order to estimate the effect of membership time on integrative complexity, we used a fixed effects linear model in which the intercept was allowed to vary by individual. Integrative complexity for each post was modeled as a function of the time since joining the forum and the word count. To best assess the within-subject effect, we limited observations to posts from members with more than one post in the dataset, yielding 1,459 observations from 280 members. In the following expression, we let *T* denote the time between joining the forum and the posting, and *W* the word count of the post:

$${IC}_{i}=\beta_{0i}+\beta_{t}T+\beta_{w}W+e_{i}$$

$$with\;\beta_{0i}=\beta_{0}+u_{0i}$$

In this model *β*_0*i*_ is the random intercept for the *i-*th individual, *β_t_* the coefficient for time to posting, *β_w_* the coefficient for word count, and *e_i_* the residuals. The results of this linear mixed model are shown in Table 1 below. Even when adjusting for individual differences in integrative complexity and each post’s word count, the time spent as a member was marginally negatively associated with integrative complexity, such that a standard deviation increase in time to posting (approximately 59 months) was associated with a .05 drop in integrative complexity (*β* = -.05, *t*(170.35) = -1.78, *p* = .07, 95% *CI* [-0.12 – 0.00]). The longer the author of a post had been a member of the forum, the less integratively complex their writing tended to be.

#### Number of posts and time to post

In Model 3, we integrated the two previous models and simultaneously estimated the relationship of both time to posting and number of posts with the dependent variable of integrative complexity (*n* = 1,459 from 280 members). Paralleling our prior analyses, the model estimated fixed effects of each post’s author to account for individual differences in integrative complexity and adjusted for word count. In this model, the between-subjects independent variable of number of posts remained significantly negatively associated with integrative complexity (*β* = -.08, *t*(119.97) = -2.38, *p* = .01, 95% *CI* [-0.15 – 0.02]). Time to post within-subjects, on the other hand, was no longer associated with complexity (*β* = -.01, *t*(177.40) = -0.52, *p* = .59, 95% *CI* [-0.08 – 0.05]).

Finally, we performed two sensitivity analyses to test whether the effect was driven by outliers in our skewed independent variable. First, we excluded outliers from the number of posts variable; for example, a small number of “super-posters,” individuals who are exceptionally active on the site, might be similarly exceptional ideologically or in their integrative complexity. We excluded all participants who were not within the median 95% of the distribution of number of posts: less than 4 and greater than 31,396 posts. Using this subset of 1,385 observations, our findings from Model 3 held; the relationship between number of posts and our dependent variable increased in strength (*β* = -.12, *t*(104.18) = -2.25, *p* = .02, 95% *CI* [-0.23 – 0.02]). The effect remained small, with 8,331 more posts associated with a .12 decrease in integrative complexity. Next, we analyzed Model 3 with a trimmed range of the number of words per post (less than four and greater than 608). Again, number of posts was associated with less integrative complexity (*β* = -.05, *t*(119.76) = -1.99, *p* = .04, 95% *CI* [-0.10 – -0.00]).

## Discussion

Across the three models tested, engagement was associated with less integrative complexity. In Model 3, when both measures of engagement were included, time to post was no longer significant but the estimate remained very similar. Although statistics did not suggest problematic multicollinearity (time to post *VIF* = 1.27), the estimate increased from -.05 to -.01, bringing the effect to insignificance. In sum, these results suggest that the more people have engaged with the forum, the more their cognition and writing presents as one-sided and polarized. These findings are strengthened by their in vivo, naturalistic data, increasing external validity. In addition, the fixed effects models are robust analyses that account for individual differences and the word count of each post. However, it is important to note the limitations of these analyses. These effects are very small; as stated above, a one standard deviation increase in engagement measures is associated with a fractional decrease in integrative complexity which ranges from one to seven. In addition, this evidence is strictly correlational, limiting the causal inferences that can be drawn. Finally, linguistic changes could also be a function of increased mirroring behavior resulting from group cohesion. In the subsequent analyses of LSM, we will address this final concern by examining whether writing style assimilates over time and engagement with the forum.

### Language style matching

In a series of hierarchical linear regressions, we sought to test whether engagement was associated with group cohesion. We modeled LSM as a function of our engagement measures, paralleling our analyses of integrative complexity. In each fixed-effects model, a unique intercept was estimated for each member in order to account for individual differences in language style and adjusted for word count. In Model 4, our predictor of interest was standardized number of posts, analyzed between subjects (*N* = 1,891). Number of posts was associated with less LSM (*β* = -.03, *t*(271.66) = -3.59, *p* < .001, 95% *CI* [-0.05, -0.02]). Members who wrote more posts on the forum tended to write more distinctly than people who wrote fewer posts, even when adjusting for word count and individual differences (see Table 2).

**Table 2:** Language style matching as a function of number of posts and time since joining the forum.

	*β*	*95% CI*	*p*
Model 4			
(Intercept)	.61	0.60 – 0.64	< 0.001***
Number of Posts (std)	-0.03	-0.05 – -0.02	< 0.001***
Words (std)	0.10	0.09 – 0.12	< 0.001***
Random Effects			
σ^2^	0.04		
τ_00_	0.01		
ICC	0.28		
Marginal R^2^ / Conditional R^2^ = 0.186 / 0.410			
Model 5			
(Intercept)	0.62	0.60 – 0.64	< 0.001***
Time to Post (std)	-0.02	-0.04 – 0.00	0.07 †
Words (std)	0.09	0.08 – 0.11	<0.001
Random Effects			
σ^2^	0.04		
τ_00_	0.01		
ICC	0.26		
Marginal R^2^ / Conditional R^2^ = 0.165 / 0.380			
Model 6			
(Intercept)	0.62	0.60 – 0.64	< 0.001***
Time to Post (std)	-0.01	-0.03 – 0.02	0.52
Number of Posts (std)	-0.02	-0.05 – -0.00	0.029 *
Words (std)	0.10	0.08 – 0.11	< 0.001***
Random Effects			
σ^2^	0.04		
τ_00_	0.01		
ICC	0.25		
Marginal R^2^ / Conditional R^2^ = 0.183 / 0.388			

Note: Random effects refer to intercepts estimated for each member.

† p< .1

* p < .05

** p < .01

*** p < .001.

In Model 5, to best assess the within-subject effect of time to posting, we limited observations to posts from members with more than one post in the dataset, yielding 1,459 observations from 280 members. Standardized time to posting was also associated with less LSM (*β* = -.02, *t*(228.33) = -1.77, *p* = .07, 95% *CI* [-0.04, 0.00]), while other estimates remained approximately the same.

In our final model, both of our measures of engagement were included in a fixed-effects model with our sample limited to the same subset of authors with multiple posts (*n* = 1,459). In this model, number of posts remained associated with less LSM (*β* = -.02, *t*(164.96) = -2.209, *p* < .001, 95% *CI* [-0.05, -0.02]), while time to posting was no longer associated with less LSM (*β* = -.01, *t*(229.37) = -0.63, *p* = .07, 95% *CI* [-0.04, 0.00]). This effect was significant, but very small, with an increase of 8,331 posts associated with a .02 drop in LSM.

## Discussion

In this series of hierarchical linear regressions, we found that engagement was negatively associated with LSM. In other words, the more that members engaged with the community, the more distinct from the group their writing was. This effect was small, but consistent across the three models. This suggests that cohesion between individual members and the group––at least as indexed by markers of linguistic similarity––was decreasing with time and number of postings. One possibility is that new members try to assimilate to the culture of the forum, and as members become more accustomed to the group or attain more status within it, they differentiate themselves from the crowd using more distinct language.

Perhaps more important than speculating on the small effect’s meaning on its own, the findings paint an interesting picture for integrative complexity. As outlined in our literature review, integrative complexity has been shown to decrease while experiencing Groupthink and deindividuation. However, as style matching decreased with engagement, signifying less cohesion, we can safely deduce that the similar drop in integrative complexity is not indicating Groupthink. Instead, it represents ideological polarization increasing with forum engagement.

Although the data are correlational and the effect sizes are generally small, the results also discount an important alternative explanation—that changes in integrative complexity are superficial artifacts of adopting the common linguistic style of the forum. For example, if simple declarative sentences—low in integrative complexity—were the cultural norm of the forum, participants’ increased style matching could explain the effects in Models 1–3. In fact, LSM and integrative complexity were strongly correlated (*r* = .54, *p* < .001), further challenging this alternative hypothesis; if acculturation to the website’s language were associated with simpler language, we would expect a negative correlation. Instead, engagement does not predict adopting the forum’s linguistic norms, therefore changes in integrative complexity are not artefacts of group cohesion.

## General discussion

Amidst mounting concern over the role of the internet in fueling political polarization [39], we examine how online extremist groups may intensify one’s ideological viewpoints. Ideological extremism and reduced tendencies to view issues as complex and multi-faceted may contribute to political polarization and antipathy between ideologically opposed groups [28]. Due to social processes that are especially pronounced in online forums, including ostensible unanimity and anonymity, extremist online groups may serve to further radicalize their members and homogenize their ideology. In this research, we take a preliminary step to address how membership within these online communities affects cognition and ideology. We found that as users became more engaged with a forum––indexed by their number of posts as well as their time since joining––their posts reflected lower integrative complexity. This indicates that cognition may become more conflict-oriented, one-sided, and less nuanced as engagement with the forum increases.

This change in cognition occurs alongside a decrease in Language Style Matching; as engagement increased, members used a more distinct linguistic style. Past research has found that as an online group becomes more cohesive, the language style of its members becomes more similar [35]. Among members of Stormfront, LSM decreased, suggesting three options. First, cohesion and group identification may actually decrease with engagement. However, as we measure engagement as both time and number of postings, it seems unlikely that people continued to post over time if they were feeling less involved. Second, past research has analyzed smaller groups, in which these much smaller groups had more direct communication and collaboration among members. Some of the proposed reasons why LSM increased in small groups may not apply to large groups like Stormfront, which boasted over 330,000 members at the time of data collection. For example, mirroring others’ phrasing and sentence structures—which happens with social identification [35]—may increase LSM in smaller groups, but fail to have a noticeable impact in larger ones unless those turns of phrase are widely used. Finally, engagement may lead members in a large group to seek a distinctive way of expressing similar, extreme ideas within the community to stand out from the crowd. Some research suggests that after initially adopting the linguistic style of the forum, familiarity and experience with an online community may subsequently let the author’s personal voice to come to light [40]. Instead of engagement predicting cohesion with the group, it could predict taking a vocal, leadership role within the crowd.

This research benefits from a large sample size of in vivo naturalistic observations. By virtue of examining individuals’ actual posts to an online forum, the data used in this research are precise logs of real behavior. As a result, they are relatively uncontaminated by demand characteristics of experimentation or bias. As outlined above, studies have shown that integrative complexity reflects genuine changes in cognition, with genuine consequences for diplomacy, decision-making, and war. Given the use of an automated coding tool, the effects should be readily replicable with new data from comparable spaces or for new applications. For example, it would be interesting to test whether similar patterns emerge as a function of engagement with an online forum associated with viewpoints of the “radical left” (e.g., eco-terrorism groups).

At the same time, this research comes with important limitations. For example, although the sample size was large relative to other social psychological studies, it is a small, unique sample compared to other big data approaches. With regard to our independent variables, although we tried to approximate engagement using two separate variables, neither was a perfect way of capturing the construct. The number of posts variable does not vary within individual, therefore, when using that measure it is impossible to estimate whether each person’s cognition becomes simpler over time or if these are stable differences of those who more dedicated to the forum. Accordingly, some research suggests that “transmitters”––those who disseminate their views––hold more extreme views than those who do not [41]. Although ideally we would analyze integrative complexity as a function of how much time has been actively spent on the forum, those data were not attainable. Instead, we used time between joining the forum and each posting as another indicator of engagement, but this, too, is an imprecise measure of the intended construct of time spent on the forum. In future research, it will be important to replicate these results with more direct measures of engagement (e.g., self-report).

The individual and social functions of integrative complexity should also be further explored. Research suggests that there may be benefits to speaking in a simple, one-sided manner. In a study of film awards, winning films tended to have lower integrative complexity than films that lost [42]; simple language may convey more appealing or relatable emotionality. In American presidents’ speeches, Jordan, Sterling, Pennebaker and Boyd found that linguistic markers of analytical, complex thinking have decreased steadily over the course of American history [43]. They point to an increase in rhetoric in its stead, which may be increasingly appealing to modern audiences. In fact, presidents tend to show less complexity when trying to connect with audiences on the campaign trail compared to after they take office [44]. In an ideological online forum, posts with simpler language may be more appealing to other members on the forum, and may be reinforced by more positive reactions from peers and greater status assigned to the poster [45]. On a group level, less integrative complexity may increase feelings of unity and solidarity in others, which are particularly valued within a group in times of conflict [29]. Future research should use experimental methodologies to tease apart extremist forums’ radicalizing effects from the intragroup and interpersonal benefits of simpler posts.

The current findings indicate that engagement in a white supremacist forum is associated with reduced integrative complexity over time. The results provide valuable insights for understanding cognition and persuasion in vivo within homogeneous groups. Future research should continue to build on these insights to better understand the social consequences of online ideological communities in hopes of shedding light on the processes that may contribute to growing ideological polarization and, ultimately, help upend it.
